# Immunologic profiling of adenoid cystic carcinoma (acc)

**DOI:** 10.1186/2051-1426-3-S2-P105

**Published:** 2015-11-04

**Authors:** Vishwajith Sridharan, Xiaoyun Liao, Nicole G Chau, Robert Haddad, Adel El-Naggar, Gordon J Freeman, F Stephen Hodi, Scott J Rodig, Glenn Dranoff, Jonathan D Schoenfeld

**Affiliations:** 1 Harvard Medical School, Boston, MA, USA; 2 Dana-Farber/Harvard Cancer Center, Boston, MA, USA; 3 Dana Farber Cancer Institute, Boston, MA, USA; 4 MD Anderson Cancer Center, Houston, TX, USA; 5 Novartis Institutes for Biomedical Research, Cambridge, MA, USA

## Background

Adenoid cystic carcinoma (ACC) is among the most common malignant salivary gland tumors. Approximately 50% of patients develop distant metastases and up to one third die within 2 years. Chemotherapy provides limited benefit, and is not associated with a survival advantage. Radiotherapy is standard for localized disease, and is also commonly used for palliation of symptomatic metastases. Little is known about endogenous immune responses directed against ACC at baseline, or following conventional treatments. Therefore, we evaluated ACC specimens for infiltrating immune cells and expression of immune checkpoint ligands PD-L1 and PD-L2 and their receptor, PD-1, to guide future investigations of immunotherapy in ACC patients.

## Methods

We retrospectively obtained whole-tissue slides from ACC patients, and prospectively collected serial tissue, serum and saliva samples from one patient treated with concurrent chemoradiation therapy with residual tumor after treatment. Hematoxylin/eosin and immunohistochemical staining was performed using immunologic markers including: CD4, CD8, FoxP3, PD-1, PD-L1, and PD-L2. Multiple areas from stained slides were evaluated and scored by a pathologist. We also evaluated the patient treated with chemoradiation for potential anti-tumor antibody responses before and after treatment using ProtoArray Immune Response Profiling (Invitrogen).

## Results

Tissue from 28 primary and metastatic ACC deposits were obtained from 20 patients. Most tumors demonstrated only mild/modest numbers of infiltrating immune cells (< 100/HPF, n=18; 64%); in the majority of other tumors, infiltrating immune cells demonstrated high levels of PD-L1 expression. ACC cells did not express PD-L1. Seventeen ACC deposits demonstrated PD-L2 expression (61%). PD-L2 was expressed in 53% of primary lesions (n=9), and 67% of metastatic deposits (n=8). Comparing the ACC tumor before and after chemoradiation, we found increased CD8+ T cells (34 compared with 110/HPF), and decreased FOXP3+ regulatory cells (104 compared with 33/HPF). After chemoradiation, proteomic analysis revealed increased titers of multiple antibodies in both serum and saliva directed against potential tumor antigens.

## Conclusions

We profiled the immunologic microenvironment in both primary and matching metastatic ACC's. Most ACC demonstrate only mild/modest numbers of tumor infiltrating immune cells. PD-L1 expression was limited to immune cells and not ACC tumor cells. In contrast, PD-L2 was expressed on tumor cells in the majority of cases. Following treatment, a patient who received chemoradiation demonstrated increased CD8+ T cells, decreased FOXP3+ regulatory T cells, and potential evidence of a more robust anti-tumor antibody response. These data suggest that PD-1 inhibition, potentially in combination with conventional treatments, could represent a promising treatment strategy for patients with ACC.

